# Land cover transformation in two post-mining landscapes subjected to different ages of reclamation since dumping of spoils

**DOI:** 10.1186/2193-1801-3-702

**Published:** 2014-11-28

**Authors:** Effah K Antwi, John Boakye-Danquah, Stephen B Asabere, Kazuhiko Takeuchi, Gerhard Wiegleb

**Affiliations:** United Nations University- Institute for the Advanced Study of Sustainability (UNU-IAS), Tokyo, Japan; The University of Tokyo, Todai Institutes for Advanced Study, Integrated Research System for Sustainability Science (IR3S), Tokyo, Japan; School of Environment and Sustainability, University of Saskatchewan, Saskatoon, SK S7N 5C8 Canada; Faculty of Environmental Sciences, Department of Geosceinces, Technische Universitaet Dresden, Dresden, Germany; Environmental Science and Process Engineering, Department of General Ecology, Technical University of Brandenburg (BTU), Cottbus, Germany

## Abstract

Transformation of natural land cover (LC) into modified LC has become inevitable due to growing human needs. Nevertheless, landscape transformational patterns during reclamation of mine damaged lands remain vague. Our hypothesis was that post-mining landscapes with different ages since dumping become more diverse in LC transformation over time. The aim was to study the impact of landscape reclamation on land cover changes (LCC) in two post-mining landscapes. Land cover maps of 1988, 1991, 1995, 1998, 2000 and 2003 were produced from LANDSAT TM images of Schlabendorf Nord and Schlabendorf Süd and used to survey the changing landscape. Change detection extension was used to identify changes among land cover types (LCTs). Detrended correspondence analyses (DCA) ordination technique (CANOCO) aided study of similarity among LC distribution. Soil pH analysis was carried out to study effect of soil and climate conditions on LCC. The results show that visible patterns of increase and decrease in the LCTs occurred in both landscapes. Given two post-mining landscapes subjected to different ages of reclamation, clear differences in vegetation growth and LCC pattern would occur. At early stages of restoration, LCTs often have unstable conditions and experience more acute transformation depending on the level of land use intensity in space and time. LCCs were mostly due to progressive and reversed succession. Due to variation in post-mining landscape soil conditions, soil treatment during reclamation should be site specific. The comparative analysis of LCCs in Schlabendorf provides a framework for prioritizing land use planning options for sustainable management of post-mining landscapes in temperate ecosystems.

## 1 Introduction

Anthropogenic disturbance such as surface coal mining is particularly rigorous in nature. Regardless of the method used and the resources available, it affects land cover (LC) at the site of extraction (Haigh 2000); leaving behind multiple damages that stretch over a wide range of land. Continuous monitoring of mining induced land cover change (LCC) is essential for sustainable mine management. Particularly, it helps to identify the impact of mining on land cover types (LCTs) and to provide the needed mine closure and reclamation measures (Demirel et al. 2011).

Ecosystems undergo long periods of natural succession and it could take hundreds of years for full recovery to occur, if it does occur at all. Human mediated restoration measures are often needed to accelerate succession processes (Groninger et al. 2007; Hendrychová 2008). Reclamation of mine-damaged lands is feasible but it may be a slow or comparatively fast process depending on the extent of damage. Therefore reclamation schemes that consider some kind of human intervention could be adapted to accelerate restoration processes and reinstate the ecological integrity of damaged landscape, giving it a defined after use. Bradshaw (1999) reported a successful study by Western Australia mining company, Alcoa, who stockpiled and reused the topsoil to restore an area after mining. Knoche et al. (2000) ascertained that ecosystems in Lower Lusatia gradually shift from a state of geochemical-dominated process to a mode of increasing biological control.

Haigh (2000) ascertained that high acidity and high concentration of aluminums in coal-mining areas are often responsible for land degradation (loss of vegetation cover). Post-mining reclamation schemes are ultimately done to help degraded lands to be productive again; and this mainly involves re-establishment of vegetation cover. For successful post-mining landscape reclamation, sustainable practices such as management and replacement of top soil should be vital during moving and storage phases of excavation (Nova Scotia Environment 2009). This eventually provides the condition favorable for the establishment of a self-sustaining, succession-based vegetation cover through the application of native seed mixtures and plant materials (Nova Scotia Environment 2009).

One of the main goals of the state of Brandenburg government in the Lusatia post-mining landscape reclamation was to enhance the production of biomass for energy use (obtain a share of 3% of the primary energy consumption from biomass) on arable set-aside and post-mining land (Bungart and Hüttl 2001). Short-rotation plantations were planted as energy forests that served the purpose of bioenergy or wood fuel production on recultivated land.

In landscape ecology, vegetation is considered as mosaic of patches with unique landforms, species composition and disturbance gradient (Ravan et al. 1998; Antwi et al. 2008). Visualizing and analyzing the landscape mosaic requires technological innovations which are mainly found in geospatial technologies including multispectral Remote Sensing (RS) and Geographic Information Systems (GIS). Launching of the LANDSAT sensor in 1972 aided the application of remote sensing for estimating terrestrial plant primary production based on Normalized Differential Vegetation Index (Young and Harris 2005). It is now possible to depict the assemblage of dominant growth forms of plant species with common habitat to identify vegetation and LC units which are essentially missing during field survey due to limitations in sampling technique (Latifovica et al. 2005).

Ravan et al. (1998) used GIS to map out disturbance zones in natural ecosystems to investigate its impact on primary production and biodiversity. Gould (2000) applied remote sensing to detect patterns of vegetation and variations in biodiversity at the mesoscale. At different spatial scales, it has become possible to map the discontinuous distribution LCTs by using LANDSAT in GIS environment (Edenius et al. 2003; Cohen and Goward 2004).

LC distribution may differ or show similarity due to the land use (LU) intensity, the age of LCT and the type of LC. The level of similarity can be monitored using detrended correspondence analysis (DCA). DCA gives the best performance among all tested ordination techniques and provides effective approximation solutions to ordination problems (Hill and Gauch 1980; Palmer 2010). Ter Braak (1996) found that in DCA, sites are represented by points and each site is located at the center of gravity of the species that occur there.

The importance of temporal and spatial LU transformations has been addressed by many LU and LCC studies (Swangjang and Iamaram 2011). LCC detection has become a central component in current strategies for monitoring LU and environmental changes (Latifovica et al. 2005). It provides better understanding of landscape dynamics during a known period of time. Through the use of spatial and temporal analyses techniques from GIS applications, change detection can provide useful applications in a wide variety of areas such as LUCs, habitat structure etc. (Latifovica et al. 2005; Rahdary et al. 2008; Antwi et al. 2014). Yang (2008) applied a satellite and airborne remote sensing technique to extract LC information that depicts present and potential landscape dynamic and structure analysis. In order to quantify the anthropogenic causes of LCC in the Lake Tahoe basin resulting from varying LU intensity on forest landscapes, Raumann and Cablk (2008), with the aid of post-classification change analysis, studied changes in four broad historical periods. They ascertained that decreased area of forest, wetlands, and shrub’ degradation corresponded with increase in built-up areas.

The research approach adopted in this study offers a trade-off between expensive ground-based LCC survey and cost effective satellite-based data analysis. The general aim of the research is to compare timely LC transformation in two post-mining landscapes which have been subjected to different ages of reclamation activities using GIS and remote sensing. The specific objectives of the research are to:

1. Identify and quantify the area of all LCTs in the two post-mining landscapes to determine the impact of surface coal mining on LC transformations and vegetation growth in the study area.
2. Study changes and similarities in LC transformation trends in two post-mining landscapes which have been subjected to different ages of land reclamation since dumping of spoils.
3. Study the interactive distribution of different habitat types and determine their relationship with environmental conditions.

The following questions were answered:

1. How does spatial and temporal LCC relate to changing environmental conditions?
2. Are the post-mining landscapes getting more diverse over time?

## 2 Materials and methods

### 2.1 Study area

The study area, Schlabendorf, is located in the Lower Lusatia region of Germany. It is regarded as an extremely disturbed post-mining landscape. Coal mining in Lusatia devastated more than 80,000 ha of land surface. However, more than 250,000 ha of the region was affected by the lowering of ground water due to extensive pumping measures prior to the opening of the mine, resulting in a groundwater deficit (7×109 m^3^) in the region (Hüttl 1998).

**Figure 1:**
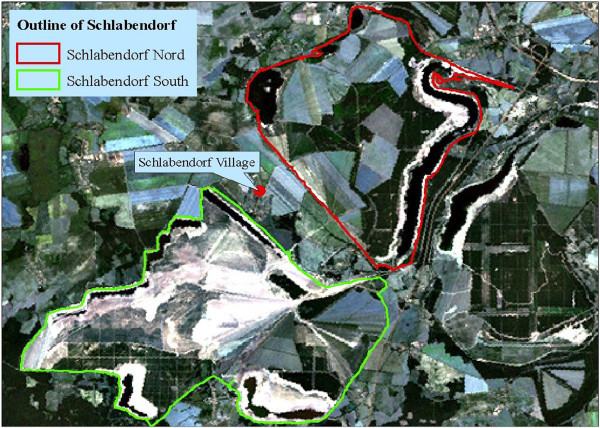
**Outlines of Schlabendorf Nord and Schlabendorf Süd on a LANDSAT TM image of 2000.**

The two post-mining landscapes are named after the village Schlabendorf (Figure 1); located between both post-mining landscapes at latitude 51.817 and longitude 13.817; dividing it into North (Nord) and South (Süd). Mining activity in Schlabendorf started in 1975 and ended 1991. Schlabendorf Süd has undergone 19 years of reclamation since dumping, and covers 3,035 ha of land surface. Schlabendorf Nord is smaller (2,585 ha), with 35 years of reclamation. Spoils in the post-mining landscape have dry habitat condition (Schultz and Wiegleb 2000) characterized by acidic sandy spoils on lignite rich tertiary sand. They have low water retention capacity and high evaporation rate high carbon content, low water capacity, low nutrient content and lack of biological activity (Grünewald 2001).

**Figure 2:**
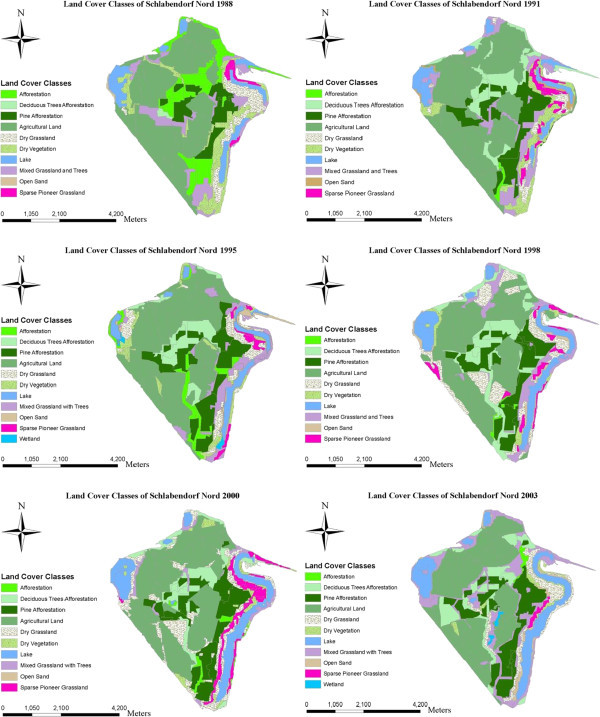
**Final classification of land cover types in Schlabendorf Nord from 1988 to 2003 showing land cover classes with and without Wetland.**

**Figure 3:**
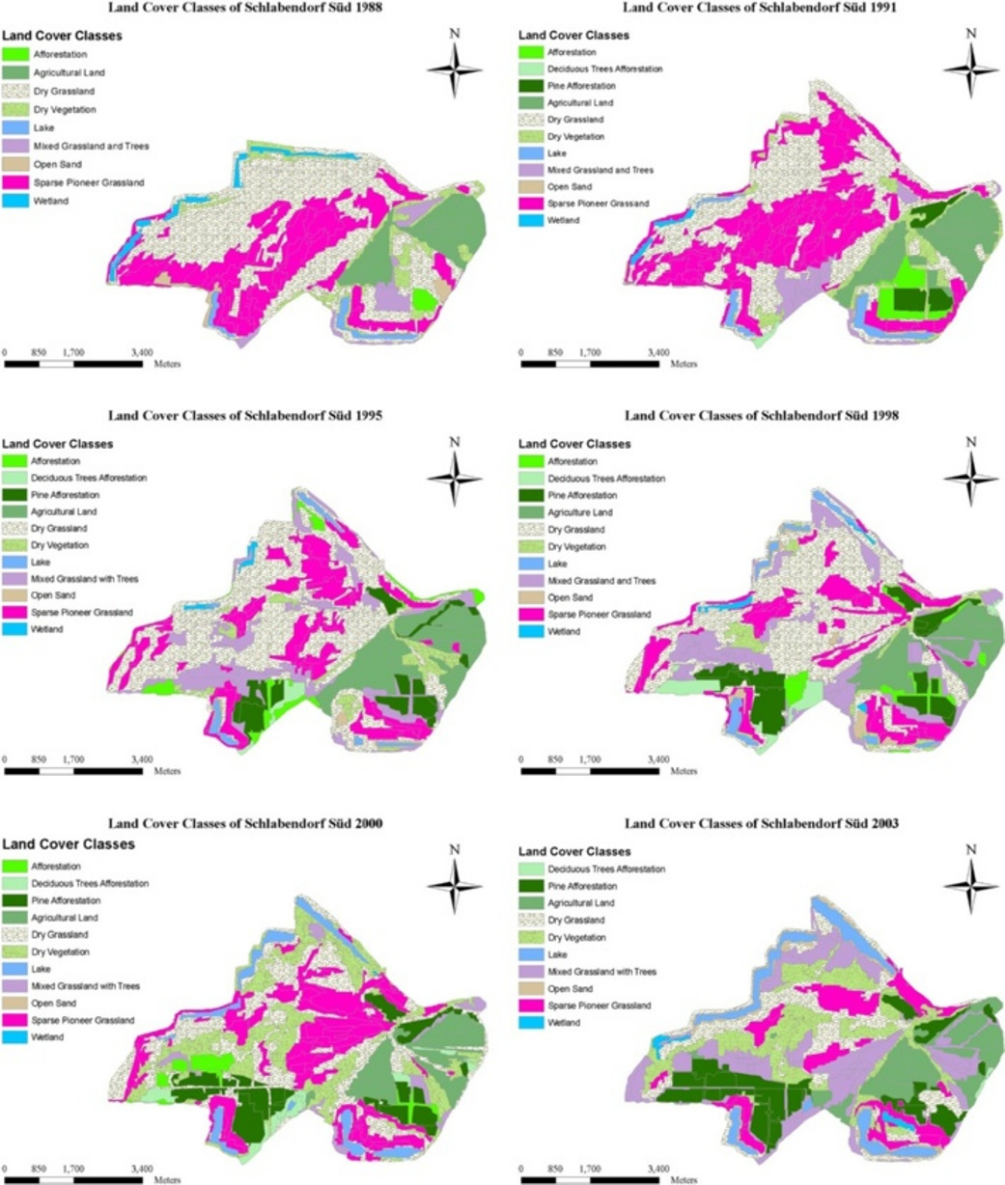
**Final classification of land cover types in Schlabendorf Süd from 1988 to 2003 showing land cover classes with Wetland present in all periods of study.**

Extensive studies on the ecological and socioeconomic problems, dominant vegetation types and ages are given by Blumrich et al. (1998), Wiegleb and Felinks (2001a, [b]), and Antwi et al. (2008). Common herbivores in the area include roe deer (*Capreolus capreolus*), wild boar (*Sus scrofa*), hare (*Lepus europaeus*), rabbit (*Lepus curpaeums*) and red deer (*Cervus elaphus*). In Figures 2 and 3 we classified the LCTs and dominant vegetation cover in both landscapes.

### 2.2 Data acquisition and spatial database

LANDSAT TM 4 and 5 and SPOT imagery of the two landscapes in the years 1988, 1991, 1995, 1998, 2000 and 2003 were used to survey the changing landscape. SPOT image was only used in 2003. Classification of LCT was based on supervised classification method using dominant vegetation type found during the years of study (Schultz and Wiegleb 2000; Antwi et al. 2008). Overall classification accuracy > 95% was attained in all map years to ensure high accuracy in the final classification output (Ravan et al. 1998). For each map ear, both landscapes and LC statistics were calculated. Also, landscape metrics were computed using Patch Analyst V-3.1 (Antwi et al. 2008). The scale of assessment was based on changes within habitat types in the landscape, i.e., habitat-mosaic (Weber et al. 2004; Young and Harris 2005).

### 2.3 Land cover change detection with the extension “Veränderung” (v3)

After the LC classification, change detection extension **“Veränderung”(v3)** was used to quantify changes from one LCT to another in both landscapes (Antwi et al. 2014). LCC maps were produced showing “negative change”, “no change” and “positive change”. All calculated changes were done using vector data model in an ArcView GIS software environment since the **“Veränderung”** works only on polygon files. “Veränderung” determines changes between two-polygon themes representing features of the same area. The two polygon themes intersect and form a union. The union polygons eventually become the output polygon theme. Non–intersecting areas are selected together with remaining records within polygons that are intersected by union (Antwi et al. 2008). Based on the selected intersecting and non-intersecting polygons, positive, negative and maintained areas are calculated from the attribute tables. Further analyses were made from the attribute table of the polygon containing the detected changes.

### 2.4 Estimation of vegetation growth (Primary Production) in Schlabendorf

Vegetation growth which is described in this study as primary production was calculated by greenness index using Normalized Differential Vegetation Index (NDVI). NDVI is the ratio of amount of energy reflectance in the near-infrared (NIR) and visible red portions of the electromagnetic spectrum, i.e., 0.72–1.10 and 0.58–0.68 mm respectively. The NDVI was calculated from LANDSAT TM 4 and 5 imagery (in 1988, 1991, 1995, 1998 and 2000) and SPOT imagery (in 2003) during vegetation period of both landscapes (formula 1) to investigate extent of vegetation growth during the reclamation. After masking the study area using ERDAS (ERDAS, 2002), near infrared (TM band 4 or SPOT band 3) and red (TM band 3 or SPOT band 2) were extracted. In the ArcGIS environment, spatial analyst extension was used for computation of biomass accumulation (NDVI) values as follows:

For LANDSAT TM images:
1

The resulting layer was made permanent. In instances where SPOT images were used, TM band 4 was replaced by SPOT band 3 and TM band 3 was replaced by SPOT band 2. NDVI values were then estimated from the attribute tables as shown in formula (2).
2

The output NDVI layer has values ranging from -1 to +1. Higher NDVI values represent a more active growth or primary production. NDVI values below 0 represent vegetation free surface to less active vegetation growth.

### 2.5 The relationship among land cover types (LCTs) from detrended correspondence analyses

Detrended correspondence analyses (DCA) were performed on LCT data in both landscapes for LC distribution analyses. DCA was used because it provides an effective approximation solution to the ordination problem (Hill and Gauch 1980). The CanoDraw CANOCO program (v-4.5) was used to plot the ordination diagram (ter Braak and Smilauer 2002) for Schlabendorf Süd and Schlabendorf Nord to display similarities among LC distributions in the ordination plane during the study period. Based on the position of the six study years on axis 1 of the ordination plane, the first DCA ordination axis correlates with gradient of increasing time of LCT from 1988 to 2003. Ordination axes two (2) explain 60.5% and 67.6% variance of the plot in Schlabendorf Süd and Schlabendorf Nord respectively, and they are both significant (Table 1). The second axis increases with increasing LC area. The further apart LCTs are from both ordination axes, the less the similarity in their spatial distribution in the landscape. LCTs close to the center of both ordination axes are those that did not undergo much transformation in area over time. Locations of LCTs at the edge are often unusual, but extreme pattern could lead to this occurrence, in which case additional data might be helpful for further clarification (Ter Braak 1996).

**Table 1:** **Ordination statistics showing eigenvalues and how the variance explain the distribution of LCTs along the primary axes of DCA at Schlabendorf Süd and Schlabendorf Nord**

	Schlabendorf Süd	Schlabendorf Nord		
	Axis 1	Axis 2	Axis 1	Axis 2
Eigenvalues	0.121	0.008	0.097	0.009
Variance explained (%)	56.9	60.5	61.6	67.6
Lengths of gradient	0.981	0.394	0.793	0.513

### 2.6 Investigating the effect of soil condition on vegetation growth

In order to investigate reasons behind failed vegetation growth along the path of mine strip in previously afforested areas, a pseudo slash shape (Ŧ) line transect was placed through areas with successful pine growth and failed pine growth. Thousand grams (g) of soil samples were taken from the topsoil layer at a depth of 0-10 cm (AG soil 1994). The samples were collected during the vegetation period at 0.50 m interval along the two horizontal and one vertical arms of the Ŧ transect for soil acidity analyses (pH). This helped to study the influence of soil pH on vegetation growth and nutrient availability.

## 3 Results

### 3. 1 Land cover distribution in Schlabendorf

**Figure 4:**
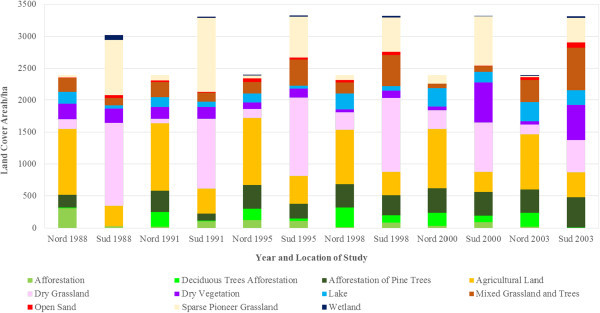
**Description of land cover distribution (in ha) in Schlabendorf Nord and Schlabendorf Süd during 1988, 1991, 1995, 1998, 2001 and 2003.**

The LC of the study areas as observed from 1988 to 2003 was classified into eleven LC types, namely afforestation, deciduous tree afforestation, afforestation of pine trees, agricultural land, dry grassland, dry vegetation, lake, mixed grassland with trees, open sand, sparse pioneer grassland and wetland. Dominant LCTs found in Schlabendorf Nord and Schlabendorf Süd are given in Figures 2 and 3. Results of LC distribution in the two post-mining landscapes during 1988, 1991, 1995, 1998, 2000 and 2003 are grouped under five major LC categories. These are: agriculture which is made of forest lands (including afforestation, deciduous trees afforestation and afforestation of pine trees), grasslands, including dry grassland, mixed grassland and trees and sparse pioneer grassland; and water bodies made up of lakes and wetlands. The last two are minor LCTs: dry vegetation and open sand. Figure 4 gives a description of LC Distribution (in ha) in Schlabendorf Nord and Schlabendorf Süd during 1988, 1991, 1995, 1998, 2001 and 2003.

#### 3.1.1 Land cover distribution in Schlabendorf Süd from 1988 to 2003

**Figure 5:**
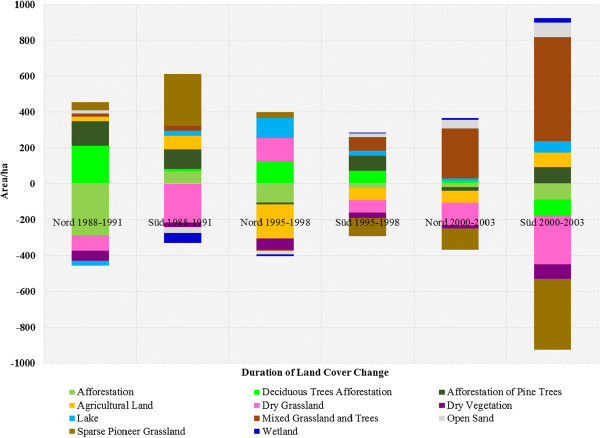
**Increase and decrease in land cover area during 1988–1991, 1995–1998 and 2000–2003 in Schlabendorf Nord and Schlabendorf Süd.**

Agricultural land generally increased between the study periods, though there were fluctuations in different years. In 1988, agricultural land occupied 321.97 ha and this increased to 399.18 ha and 442.18 ha in 1991 and 1995 respectively. However, land allocated for farming in 1998 decreased to 370.60 ha and again to 316.80 ha in 2000, though there was a marginal increase to 396.79 ha in 2003 (Figures 4, 5 and Table 2).

**Table 2:** **Land cover distribution in Schlabendorf Süd from 1988 to 2003**

Land use division (Süd)	1988/ha	1991/ha	1995/ha	1998/ha	2000/ha	2003/ha
**1. Agriculture**						
Agricultural Land	321.97	399.18	442.18	370.6	316.8	396.79
**2. Forest land**						
Afforestation	25.47	96.89	105.02	84.79	87.64	0
Deciduous Trees Afforestation	0	12.67	34.47	107.89	97.77	6.27
Afforestation of Pine Trees	0	108.14	232.5	315.03	373.32	467.54
**3. Grasslands**						
Dry Grassland	1299.55	1084.71	1222.43	1154.39	776.28	505.81
Mixed Grassland and Trees	114.63	143.26	411.91	488.22	89.89	671.3
Sparse Pioneer Grassland	868.65	1155.21	634.37	532.73	775.1	381.13
**4. Water bodies**						
Lake	53.6	80.15	47.61	76.37	169.02	232.18
Wetland	72.99	19.71	17.54	24.71	0.95	27.34
**5. Others**						
Dry Vegetation	218.11	191.03	142.73	111.77	624.18	542.8
Open Sand	45.34	12.38	29.66	49.32	3.02	82.62
**Total**	**3020.31**	**3303.33**	**3320.42**	**3315.82**	**3313.97**	**3313.78**

Forest lands in the post-mining landscape include afforestation (made up of pine and deciduous tree), deciduous tree afforestation and afforestation of pine trees. Except afforestation which occupied 25.47 ha in 1988, none of the forest LC types existed in 1988 (Figures 4, 5 and Table 2). By 2003, there was no afforestation land. On the other hand; deciduous tree afforestation generally increased from 12.67 ha in 1991 to 34.47 ha and 107.89 ha in 1995 and 1998 respectively. However, by 2000, deciduous afforestation decreased to 97.77 ha and further decreased to 6.27 ha in 2003. Since its introduction in 1991, afforestation of pine trees was the only forest land that consistently increased in all the study years. From 108.14 ha in 1991, afforestation of pine trees experienced consistent increase in area and recorded 467.54 ha in 2003. The general observation was that, while afforestation and deciduous tree afforestation were decreasing, afforestation of pine trees was increasing.

Grasslands LC distributions in Schlabendorf Süd generally involved dry grassland, mixed grassland and trees and sparse pioneer grassland. There was a general decrease in all the grasslands’ LCTs from 1988 to 2003 (Figures 4, 5 and Table 2). In 1988, dry grassland covered 1299.55 ha but this decreased to 1084.71 ha in 1991, though it increased to 1222.43 ha in 1995. However, in 1998, there was a decrease to 1154.39 ha. By 2000 dry grassland significantly decreased to 776.28 ha and again in 2003 to 505.81 ha. Mixed grassland and trees decreased from 114.63 ha in 1988 to 671.30 ha in 2003. The biggest decrease in mixed grassland occurred in 2000 when it decreased to 89.89 ha (Figures 4, 5 and Table 2).

Water bodies involved lake and wetland LCTs. While lake coverage generally increased throughout the study period, wetlands decreased (Figures 4, 5 and Table 2). At the start of the study in 1988, lake covered 53.6 ha, but by the end of the study year in 2003, lake had increased more than four times its area to 232.18 ha. The year 2000 recorded the lowest decrease in wetlands size, recording 0.95 ha. In 1988, wetlands occupied 72.99 ha, this generally decreased to 27.34 ha in 2003. Wetlands in Schlabendorf Süd showed a more unsettling distribution.

The open and dry vegetation LCTs consist of dry vegetation and open sand. In 1988, dry vegetation occupied 218.11 ha, but this decreased consistently to 191.03 ha, 142.73 ha and 111.77 ha in 1991, 1995 and 1998 respectively. In the case of open sand, between 1988 and 2003, there was an increase from 45.34 ha to 82.62 ha. It is however important to emphasize that, the years 1991 and 2000 recorded the lowest declines in open sand LCT of 12.38 ha and 3.02 ha respectively. Open sand areas are created as results of failed vegetation growth in afforested areas and locations of harvested pine trees. Others open sand areas are the results of dunes (Figures 4, 5 and Table 2).

#### 3.1.2 Land cover distribution in Schlabendorf Nord from 1988 to 2003

In Schlabendorf Nord agricultural land generally decreased in size between 1988 and 2003, from 1038.58 ha to 872.30 ha respectively (Figures 5 and Table 3). Though there was an increase in agricultural land in 1991 from the previous study year, the decrease started in 1995 and continued to 1998, recording just about 856 ha. However, in 2000, agricultural land increased to 935.47 ha, but this was not sustained as there was a further decrease in 2003 to 872.30 ha.

**Table 3:** **Land cover distribution in Schlabendorf Nord from 1988 to 2003**

Land use division (Nord)	1988	1991	1995	1998	2000	2003
**1. Agriculture**						
Agricultural Land	1038.58	1065.84	1050.05	856	935.47	872.3
**2. Forest Land**						
Afforestation	302.98	16.79	114.54	12.33	34.12	16.74
Deciduous Trees Afforestation	11.48	225.11	180.25	306.11	199.11	215.99
Afforestation of Pine Trees	200.03	334.2	372.74	363.15	386.55	364.18
**3. Grasslands**						
Dry Grassland	146.74	60.71	141.66	272.23	280.8	153.54
Mixed Grassland and Trees	221.16	238.56	182.94	178.17	69.34	349.17
Sparse Pioneer Grassland	34.05	80.12	45.02	78.37	135.9	17.56
**4. Water Bodies**						
Lake	183.11	154.29	139.73	249.61	285.33	298.96
Wetland	0	0	11.89	0	0	11.16
**5. Others**						
Dry Vegetation	244.64	189.64	100.58	40.1	63.28	44.64
Open Sand	6.91	24.65	57.28	36.7	1.35	46.71
**Total**	2389.68	2389.91	2396.68	2392.77	2391.25	2390.95

Forest LC distributions involved afforestation, deciduous tree afforestation and afforestation of pine trees. Except afforestation which decreased in size between the study periods, deciduous tree afforestation and afforestation of pine trees generally increased in size from 1988 to 2003 (Figure 5 and Table 3). In 1988, afforestation land occupied 302.98 ha. However, by 2003, this had decreased to 16.74 ha. Over the period, the highest decline in afforestation occurred in 1998 where it occupied just 12.33 ha.

Deciduous forest recorded the highest increase in size from 11.48 ha to 215.99 ha in 1988 and 2003 respectively (Figure 5 and Table 3). However, the increase in year was inconsistent. From a low base of 11.48 ha in 1988, deciduous tree afforestation increased to 225.11 ha in 1991, fell to 180.25 ha in 1995, but increased again to 306.11 ha in 1998 before decreasing again in 2000 and finally picking up to 215.99 ha in 2003. Afforestation of pine trees recorded a consistent increase in size from 1988, 1991 and 1995, i.e., 200.03 ha, 334.20 ha, and 372.74 ha respectively. In 1998 afforestation of pine trees recorded a decrease in size to 363.15 ha from the previous study year, but this increased slightly to 386.55 ha before declining marginally again to 364.18 ha in 2003.

While both dry grassland and mixed grassland and tree generally increased in size from 1988 to 2003, sparse pioneer grassland generally decreased over the same period. It is important to emphasize that, the increase recorded in both dry grassland and mixed grassland and tree were not consistent throughout the study period. For instance, in 1988, while dry grassland recorded 146.74 ha, by 1991, this decreased to 60.71 ha, though there was an increase to 141.66 ha by 1995, 272.23 ha in 1998 and 280.80 ha in 2000. In 2003, dry grassland recorded 153.54 ha, which is a decrease from the previous study year. More sparse pioneer grassland was introduced in Schlabendorf Nord in 1991 and 1998 (Figure 5 and Table 3).

Lakes and wetlands formed the main water bodies in Schlabendorf Nord. While Lake LCT was present in all the study periods, wetlands only emerged in 1995 and 2003 (Figures 4, 5 and Table 3). In 1995 the wetland area was 11.89 ha. However, by 2003 when it reappeared on the LC scene this had decreased marginally to 11.16 ha. It has to be emphasized that the location of the 11.16 ha wetland in 2003 was different from that of 1995. On the other hand, the lake generally increased from 183.11 ha in 1988 to 298.96 ha in 2003. Apart from 1991 and 1995 when the lake seize decreased, there was a consistent increase in lake size in 1998, 2000 and 2003.

Open sand and dry vegetation constitute vegetation free areas and dry vegetation LC distributions in Schlabendorf Nord. While dry vegetation decreased in size from 1988 to 2003, open sand increased in size within the same period, though in both cases the changes were not consistent (Figures 4, 5 and Table 3). From 244.64 ha in 1988, dry vegetation decreased to 189.64 ha in 1991 and to 100.58 ha in 1995. The most significant decrease occurred in 1998 (40.10 ha), though by 2000 there was a marginal increase to 63.23 ha and a final decline to 44.64 ha in 2003. Open sand on the other hand increased from 6.91 ha to 46.71 ha from 1988 to 2003. However, the year 2000 recorded a significant decline in open sand area of 1.35 ha.

### 3.2 Implication of LCCs on vegetation succession and water bodies in the post-mining landscape

#### 3.2.1 Land cover change and primary succession in Schlabendorf post-mining landscape

LCC involving primary succession generally had three main LCTs: agricultural land, dry grassland and pioneer grassland changing to different LCTs. These are discussed in three segments. Segment one spans between 1988 and 1991, segment two is between 1995–1988 and segment three from 2000 to 2003. In Schlabendorf Nord, from 1995 to 1998, agricultural lands were abandoned for either dry grassland (14.28 ha) or mixed grassland and trees (5.46 ha). On the other hand, dry grassland mainly changed to dry vegetation and grassland and trees (Table 4). While changes to dry vegetation occurred in both Schlabendorf Süd and Nord in the first segment between 1988 and 1991, changes to grassland with trees first occurred only in Schlabendorf Süd in the first segment and then in both Schlabendorf Süd and Nord in the third segment (Table 4).

**Table 4:** **Land cover changes in the post-mining landscape at different study periods**

Type of land use changes	1988 - 1991		1995 - 1998		2000 - 2003	
	Nord	Süd	Nord	Süd	Nord	Süd
**Primary succession**						
Agricultural Land to Dry Grassland			14.28			
Agricultural Land to Grassland and Trees			5.46			
Dry Grassland to Dry Vegetation	22.93	6.74				
Dry Grassland to Grassland and Trees		5.99			31.01	24.37
Pioneer Grassland - Grassland and Trees		4.29			21.93	7.23
Pioneer Grassland to Dry Vegetation						16.33
Pioneer Grassland to Dry Grassland			53.54		52.33	31.62
Open Sand to Pioneer Grassland		78.13				
Wetland to Pioneer Grassland		25.69				
Dry Vegetation to Grassland and Trees	34.60				12.33	
Lake to Grassland and Trees	4.59					
**Afforestation**						
Agricultural Land - Pine Afforestation				11.72		
Agricultural Land - Afforestation		6.51				
Agriculture Land to Deciduous Afforestation	4.55		5.04		10.13	
Dry Grassland to Afforestation		3.54				5.19
Dry Vegetation to Afforestation		12.49				
Afforestation to Pine Afforestation	23.81	97.05	15.69			49.68
Afforestation to Deciduous Afforestation	52.90		42.86			
Deciduous Afforestation to Pine Afforestation						30.18
Grassland and Trees to Pine Afforestation	26.78		5.79	21.57		15.79
Grassland and Trees to Deciduous Afforestation				6.40		
**Reversed succession**						
Afforestation to Grassland and Trees					72.65	38.36
Deciduous Afforestation to Grassland and Trees					28.42	34.76
Dry Vegetation to Dry Grassland	37.37	29.00		37.33		
Dry Vegetation to Open Sand						2.53
Dry Vegetation to Agriculture Land	12.71				37.19	
Dry Grassland to Open Sand		0.67		2.23	6.21	3.83
Dry Grassland to Agriculture Land		3.16			32.04	
Pioneer Grassland to Dry Vegetation		3.52				
Pioneer Grassland to Open Sand				1.46		3.26
Afforestation to Agriculture Land	11.21					
Pine Afforestation to Grassland and Trees					6.58	2.91
Dry Grassland to Pioneer Grassland	34.13		17.38			
Grassland and Trees to Dry Grassland				19.45		
Grassland and Trees to Agriculture			17.79			
Grassland and Trees to Agriculture Land	22.34					
**Lake formation**						
Dry Grassland to Lake		0.64	25.80	1.73	4.63	3.28
Dry Vegetation to Lake		6.18		2.23	7.5	5.07
Open sand to Lake		6.26	45.82	38.14		40.93
Pioneer Grassland to Lake		1.25		0.43	4.89	2.76
Wetland to Lake		7.80	88.25	35.27		65.61
Grassland and Trees to Lake			10.01		5.78	
Dry Grassland to Wetland				0.69		
Pioneer Grassland to Wetland				0.57		1.40
Agriculture Land to Wetland					1.20	

**Figure 6:**
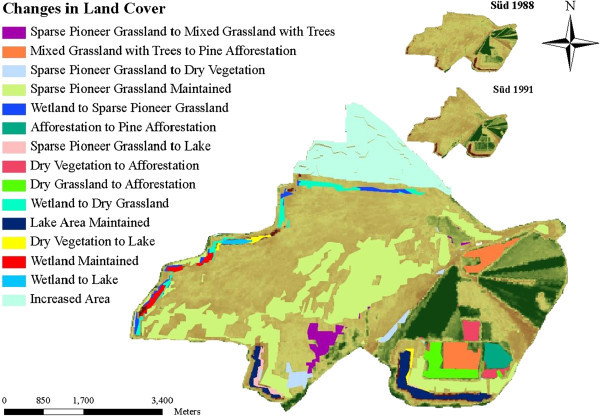
**LCC illustrations at Schlabendorf Süd from 1988 to 1991.**

In Schlabendorf, pioneer grassland, made up of mostly diaspora hay mulch, was mainly used as primary colonizer. Typically, this was seeded grassland. During the early days of succession, pioneer grassland, which was introduced to often bare areas, was replaced by grassland with trees, dry vegetation and dry grassland. The changes to grassland and trees occurred first in Schlabendorf Süd in the first segment (Figure 6). In the first segment, 4.29 ha of pioneer grassland was replaced by mixed grassland and trees, while in the last segment the replaced area came to 21.93 ha in Nord and 7.23 ha in Süd. The replacement of pioneer grassland with dry grassland on the other hand occurred only in Schlabendorf Süd in the last segment. Land cover changes from pioneer grassland to dry grassland took place in Schlabendorf Nord in both the second and third segments and in Schlabendorf Süd in the third segment only (Table 4).

**Figure 7:**
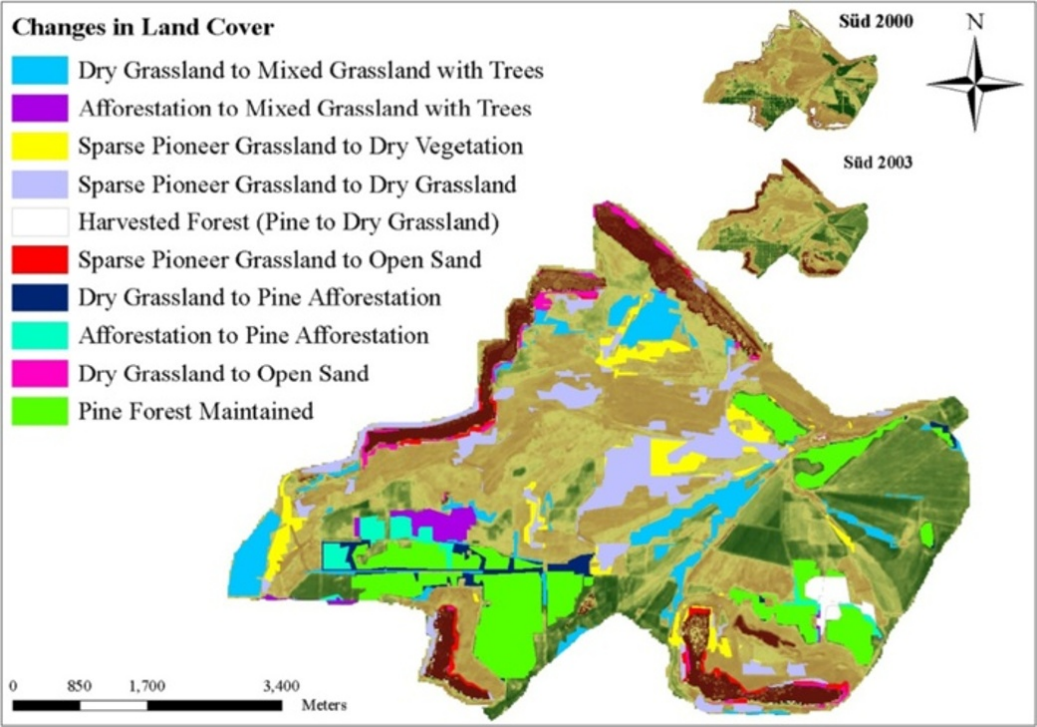
**LCC illustrations at Schlabendorf Süd from 2000 to 2003.**

Besides the above successional changes, other LCCs contributed to the primary succession stage of the post-mining landscape. These include planting 78.13 ha of sparse pioneer grassland in open sand areas in Schlabendorf Süd from 1988 to 1991. During the same period in the same landscape, 25.69 ha of wetlands area was replaced with sparse pioneer grassland (Figure 6). Other successional changes in Schlabendorf Nord include significant tree growth in the grassland (during the first and last segments) which saw the replacement of dry vegetation LCT replaced with mixed grassland and tree LCT. A portion of lake (4.59 ha) changed to mixed grassland and trees during the first segment (Table 4).

**Figure 8:**
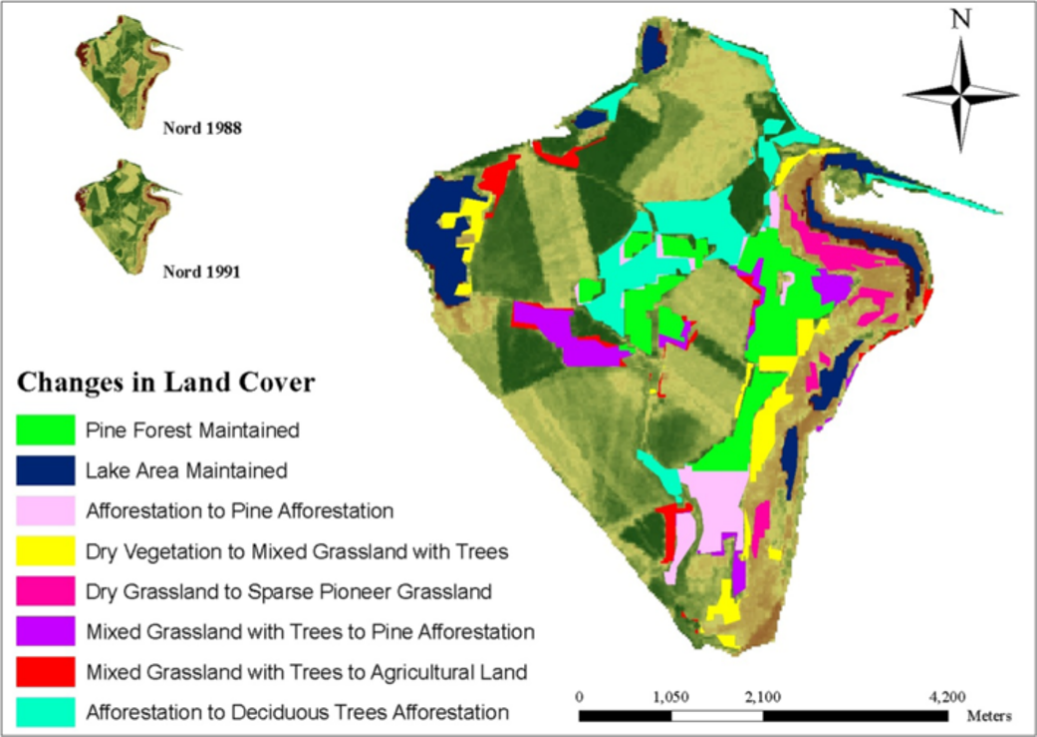
**LCC illustrations at Schlabendorf Nord from 1988 to 1991.**

#### 3.2.2 Land cover change and post-mining landscape afforestation

Most of the arable lands were abandoned for afforestation. Agricultural lands were generally replaced by afforestation of pine trees, deciduous afforestation and afforestation. In Schlabendorf Süd, afforestation from agricultural land occurred in the first segment (Table 4). However, replacing agricultural land with deciduous tree afforestation only occurred in Schlabendorf Nord in all the three segments. Apart from agricultural lands, other LCTs that contributed to forest growth are mixed grassland and trees and afforestation (Figures 6, 7, 8 and Table 4). Mixed grassland and trees contributed to the formation of pine and deciduous afforestation. The gains in afforestation of pine trees occurred in both landscapes. In Schlabendorf Nord, the gains occurred in the first segment and second segment with 523 26.78 ha and 5.79 ha respectively. In Schlabendorf Süd 21.57 ha and 15.79 ha gain occurred in the second and third segment respectively. In Schlabendorf Süd only 6.40 ha of mixed grassland and trees was lost to deciduous afforestation (Table 4).

Dry grassland and dry vegetation were both used for forest growth in the afforestation LCT. Changes from dry grassland to afforestation occurred only in Schlabendorf Süd where a change of 3.54 ha and 5.19 ha occurred in the first and third segments respectively. On the other hand, dry vegetation LCT was replaced with afforestation only in Schlabendorf Süd during the first segment, with 12.49 ha of dry grassland involved (Table 4).

Among the afforested lands, afforestation of pine trees and deciduous afforestation gained more from the losses in other LCTs. However, there were instances where deciduous afforestation also lost to afforestation of pine trees (Table 4). While other LUTs were changing to afforestation (mixed forest), afforestation often lost to afforestation of pine trees and deciduous tree afforestation. In Schlabendorf Nord, 23.81 ha and 15.69 ha of afforestation was replaced by afforestation of pine trees in the first segment and second segments. In Schlabendorf Süd, however, replacement of afforestation by afforestation of pine trees occurred in the first and last segments. In the first segment 97.05 ha of afforestation was replaced by afforestation of pine trees (Figures 6 and 8) in the last segment the coverage was 49.68 ha (Table 4).

#### 3.2.3 Land cover change and vegetation reversed succession

Reverse succession occurred mostly from changes in dry vegetation to either dry grassland, open sand, or agriculture land, while dry grassland also changed to open sand, agriculture land and pioneer grassland. Changes from dry vegetation to dry grassland mostly occurred in Schlabendorf Süd in the first and second segments from 29 ha to 37.33 ha respectively. However, in the first segment in Schlabendorf Nord 37.37 ha of dry vegetation changed to dry grassland. On the other hand, changes from dry vegetation to agricultural land occurred only in Schlabendorf Nord in the first and last segment from 12.71 ha to 37.19 ha respectively. LLCs from dry vegetation to open sand occurred only in the last segment in Schlabendorf Süd where 2.53 ha were involved (Table 4).

**Figure 9:**
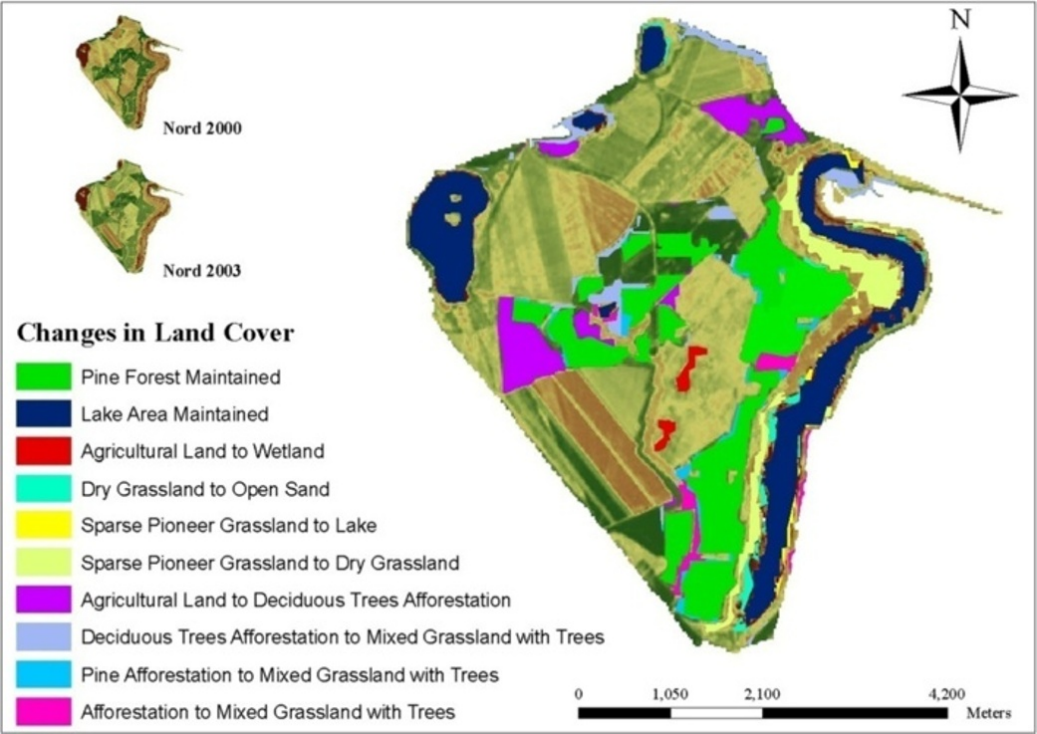
**LCC illustrations at Schlabendorf Nord from 2000 to 2003.**

Dry grassland was lost to open sand, pioneer grassland and agriculture land (Figures 8, 9 and Table 4). Changes in dry grassland to open sand mostly occurred in Schlabendorf Süd in the first, second and third segments from 0.67 ha, 2.23 ha and 3.83 ha respectively. In the third segment, 6.21 ha of dry grassland changed to open sand in Schlabendorf Nord. In the first and second segments in Schlabendorf Nord, 34.13 ha and 17.38 ha of dry grassland changed to pioneer grassland (Figure 8 and Table 4). Pioneer grassland was also lost to dry vegetation and open sand.

Changes from pioneer grassland to open sand occurred in only Schlabendorf Süd in the second and third segments (Figure 7 and Table 4). Other notable reverse successions involved changes from afforestation to agriculture land and from afforestation of pine trees to mixed grassland and trees. 11.21 ha of afforestation land changed to agriculture land only in the first segment, while 6.58 ha and 2.91 ha of afforestation of pine trees changed to grassland and trees respectively (Figure 7 and Table 4).

#### 3.2.4 Land cover change and formation of lake

**Figure 10:**
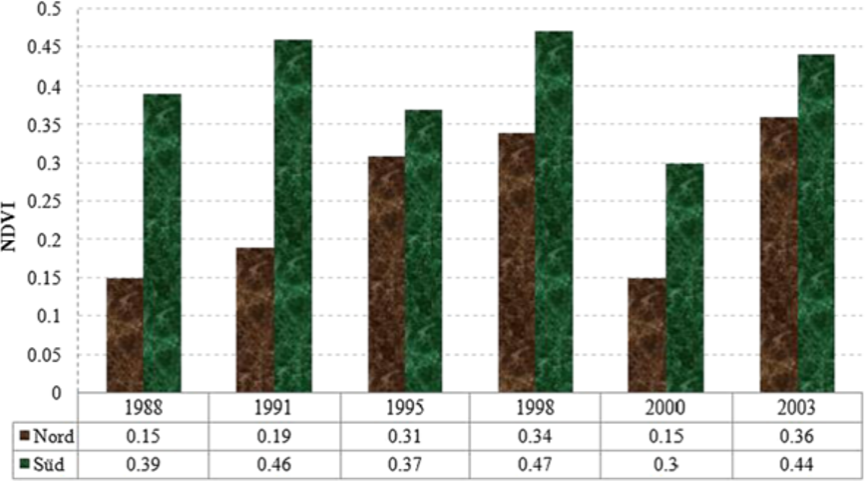
**Primary productions for Schlabendorf Nord and Schlabendorf Süd in 1988, 1991, 1995, 1998, 2000 and 2003.**

**Figure 11:**
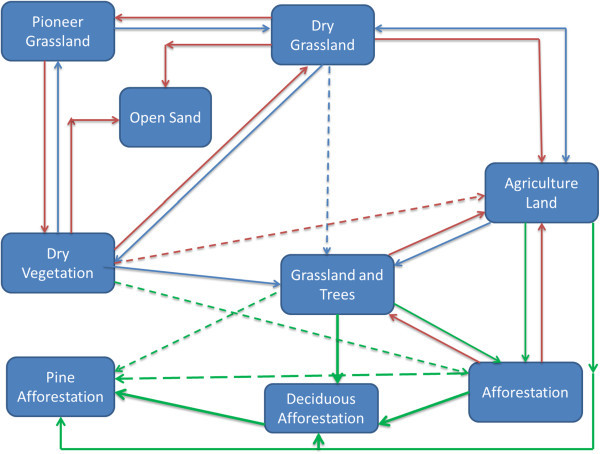
**Land cover changes and successional pathways in Schlabendorf Nord.**

The major LCTs that were converted to lake include dry grassland, pioneer grassland, dry vegetation, open sand, wetland, grassland and tree and agricultural land. In 1988, there was no wetlands in Schlabendorf Nord (Figures 6, 9, 10, 11 and Table 3). Conversion of Agricultural land to wetlands recorded the least change with just 1.20 ha involved.

### 3.3 Estimation of primary production from normalized differential vegetation index

NDVI values in Schlabendorf Nord showed greater variation in primary production from 1988 to 2003. The highest NDVI values in Schlabendorf Nord and Süd were in 1991, 1998 and 2003 (Figure 10). Unlike Schlabendorf Süd which had a largely consistent increase in NDVI values, Schlabendorf Nord had fluctuating and generally greater NDVI values. In both landscapes, the least primary production was observed in 2000. Schlabendorf Süd had a consistent increase in primary production except in 2000. In Schlabendorf Süd, though primary production decreased in 2000 to its initial value in 1988, there was a sharp increase in 2003 when the highest NDVI value was recorded. The years 1988 and 2000 recorded the least primary production.

## 4 Discussions

### 4.1 Land cover distributions and change analysis in Schlabendorf Nord and Schlabendorf Süd

#### 4.1.1 Land cover distributions and change analysis in Schlabendorf Nord

In Schlabendorf Nord, vegetation growth was mainly in the form of pioneer grassland and dry grassland changing to dry vegetation which, together with agricultural land, were changed to mixed grassland and trees. Subsequently, all the forest LCTs (afforestation, deciduous afforestation and afforestation of pine trees) were derived from mixed grassland and trees. In Figure 11 successional pathway in Schlabendorf Nord is shown. In terms of succession successes, pine forest and mixed grassland represent significant restoration success (in areas intended for recultivation) and contributed greatly to the increasing net primary production in most periods of assessment. Agricultural land also played a significant role in the forest conversion witnessed in Schlabendorf Nord, as most of the agricultural lands were abandoned for forest growth (Figure 11 and Table 4). Bungart and Hüttl (2001) found that the decline in agricultural land enabled forest recovery in Schlabendorf. Sustainable LU and an increase in forest area could occur if forest conservation, agricultural abandonment, and forest-cutting cycles are managed specifically to increase forest extent (Drummond and Loveland 2010). Nevertheless this assertion strongly depends on intended reclamation goal. Interestingly, agricultural land emerged as the most stable LCT in Schlabendorf Nord. For instance, in 1988, 1991 and 1995 (Figure 4 and Table 4), agricultural land did not undergo any significant changes. Thus, though much of its area changed to other LCTs, it also received losses from other LCTs. This also confirms the pattern that emerged in the ordination plane as agricultural land was the most centrally placed (Figure 13).

In Schlabendorf Nord, there was barely any recorded transition between open sand and pioneer grassland (Figure 11). This suggests that the vegetation growth in the old post-mining landscape has passed the level where primary colonizers grow from bare land and sand dunes. On the other hand, some vegetated areas, e.g., dry grassland and dry vegetation, became open sand, giving an indication of reverse succession. It was notable that agricultural land contributed significantly to reverse succession as LCTs such as afforestation, mixed grassland and trees, dry vegetation and dry grassland areas were turned into agricultural land (Figure 11 and Table 4).

Most of the afforestation land was lost to deciduous afforestation and afforestation of pine trees. This is visible from the continuously increasing size of afforestation of deciduous trees and decreasing size of afforestation. Any increase in deciduous afforestation and afforestation of pine trees LC corresponded to decreased afforestation LCT in the same year of study. This dissimilarity as well existed between afforestation of deciduous trees and dry vegetation. This explains why they are positioned at the extreme opposite ends of the ordination plane (Figure 13). The dissimilarity in distribution pattern of afforestation and deciduous tree afforestation LCTs is due to the fact that afforestation was made up of a mosaic of free growing pine and deciduous trees (mixed forest), which was gradually cultivated predominantly with deciduous trees and partly with pine trees (Table 4).

The pattern of distribution of afforestation was similar to that of dry vegetation in most of the period of study (Figures 4, 13 and Table 3). But since afforestation generally decreased at the end of 2003, increase in the dry vegetation in this case supports reverse succession. This inference is made from the general increase in dry vegetation area but not strictly linked to decreased size of afforestation.

Some LC distribution patterns were similar or close to, but not dependent on others. An example in Schlabendorf Nord is where mixed grassland and open sand largely had similar LC distribution for the most part of the study except the variation in 1991.

#### 4.1.2 Land cover distributions and change analysis in Schlabendorf Süd

**Figure 12:**
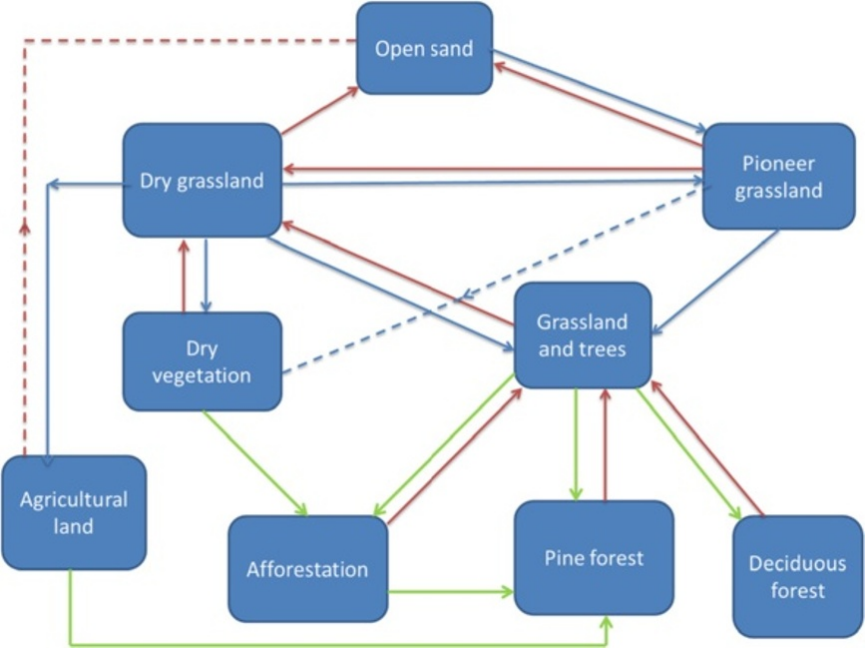
**Land cover changes and successional pathways in Schlabendorf Süd.**

**Figure 13:**
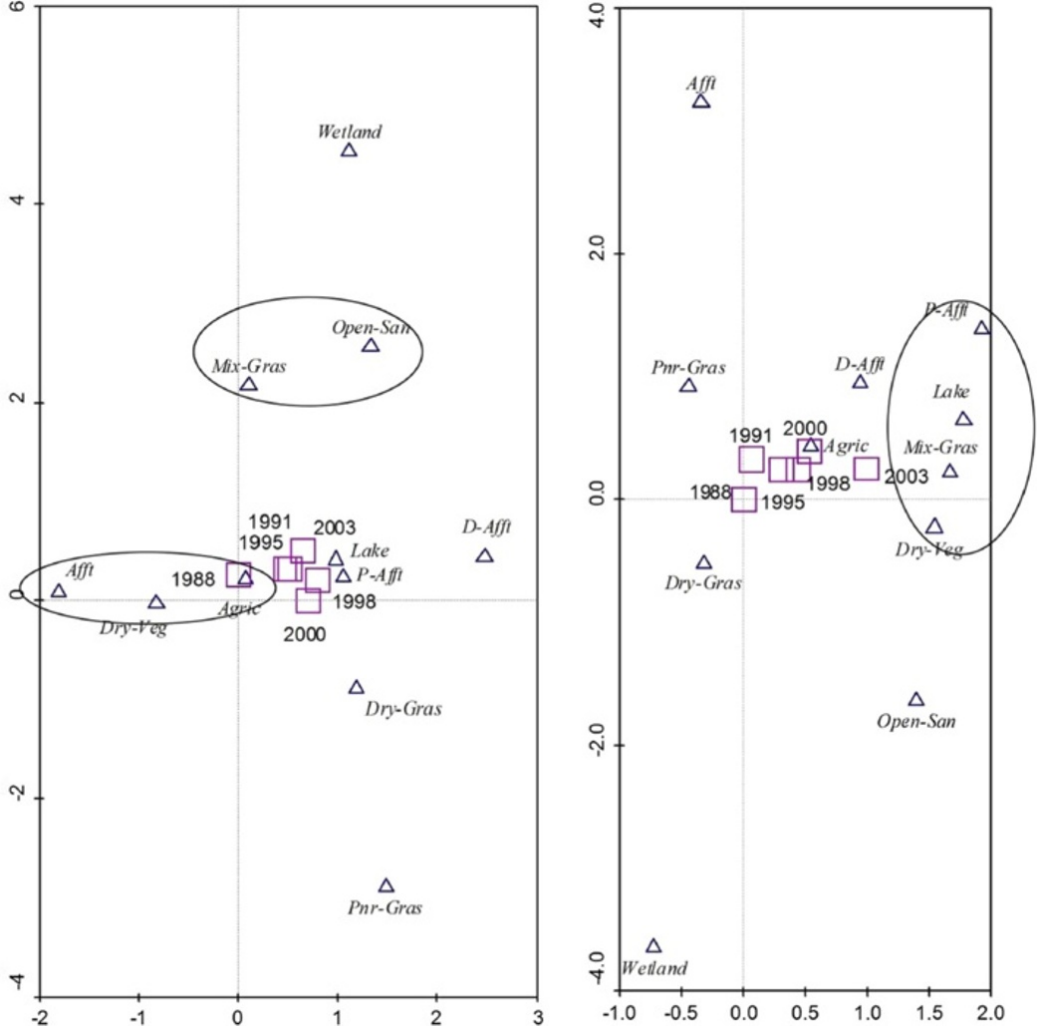
**DCA ordination plot for land cover distribution at Schlabendorf Nord (Left) and Schlabendorf Süd (Right) with untransformed data.** Legend: P-Afft - represents afforestation of pine trees, Afft - afforestation, D-Afft - deciduous trees afforestation, Dry-Veg - dry vegetation, Agric - agriculture land, Mix-Grass - mixed grassland and trees, Pnr-Grass - sparse pioneer grassland, Dry-Grass - dry grassland, Open-Sand - open sand, Wetland - wetland, Lake - lake.

In Schlabendorf Süd, the transition in LCTs between the lower vegetation cover (e.g., pioneer grassland, dry vegetation, and dry grassland) and forest land is mixed grassland and trees. The succession pathways towards afforestation involve conversions of pioneer grassland and dry grassland to grassland and tress; mixed grassland and trees were then converted to afforestation, afforestation of pine trees or deciduous forest. In addition, dry vegetation and agricultural land also contributed to forest land gains (Figure 12 and Table 4). The successional pathway of Schlabendorf Süd is shown in (Figure 12). LC distribution patterns involving afforestation of pine trees, lake and mixed grassland in Schlabendorf Süd were mostly similar, though this similarity does not indicate any ecological interdependence or process. They lie close to each other on the same side of the ordination plane because they generally increased in size during the study period (Figure 13 and Table 4). Pine forest and mixed grassland represent significant restoration success (in areas intended for forest growth) and contributed greatly to the increasing net primary production in most periods of assessment (Figure 12).

While in the case of Schlabendorf Nord, deciduous forest was changed to both afforestation and/or pine forest, in the case of the Schlabendorf Süd, deciduous forest rather lost to grassland with trees, indicating a reverse succession. In addition to this, both pine forest and afforestation also lost to mixed grassland with trees. Other notable forms of reverse succession involved changes from agricultural land, dry grassland and pioneer grassland to open sand (Figure 12 and Table 4). For instance, dry grassland cover experienced a reverse of the parallel transformation observed with regard to afforestation of pine trees, lake and mixed grassland throughout the study period. This is a typical case where reverse succession occurred in the landscape at Schlabendorf Süd. These transformations show that in Schlabendorf Süd there were concurrent back and forth gains and losses in LCTs. These transformations suggest succession and reverse succession among LCTin most cases (Felinks 2000). This is confirmed by the fact that, for most periods studied in Schlabendorf Süd, LC distribution pattern of agricultural land and deciduous tree afforestation were similar, though some variation occurred in both vegetation types in 1998 and 2003 (Figure 13 and Table 4). An increase in one corresponded to an increase in the other (Table 2). Here portions of deciduous trees in a naturally growing area with pine trees were replaced with afforestation of pine trees and others also emerged as mixed grassland with trees in 2003. Agricultural land did not change significantly.

As exemplified in the ordination plane, most LCTs in Schlabendorf Süd (4 out of the 11) were located on the left side of the first ordination axis as compared to 2 out of 11 LCTs in Schlabendorf Nord (Figure 13). The location of afforestation, pioneer grassland and dry grassland at Schlabendorf Süd implies decreasing LC area over the study period that favours reverse succession. The greater part of the sown grassland failed. Change detection results reveal that a greater part of the area that was lost in pioneer grassland became open sand (Table 4). Thus reverse succession occurred much more in Schlabendorf Süd as compared to Schlabendorf Nord.

#### 4.1.3 Comparative land cover distribution and change in the two post-mining landscapes

While in Schlabendorf Nord afforestation was replaced by afforestation of pine trees and deciduous afforestation, in Schlabendorf Süd afforestation was replaced by only afforestation of pine trees (as forest land) and mixed grassland and trees. In Schlabendorf Süd, it was more common for forest to be lost to grassland, but in the Schlabendorf Nord this was less common. In Schlabendorf Süd, all the forest lands were lost to grassland, but in Schlabendorf Nord, only afforestation was lost to grassland, suggesting more reverse succession in Schlabendorf Süd (Figures 11 and 13). Until recently, in Schlabendorf, seeded grassland were used as primary colonizers to reclaim the mine-damaged land. On the other hand, reverse succession occurred in both landscapes. An interesting trend of reverse succession was the frequent change of dry vegetation/dry grassland/sparse pioneer grassland to open sand, open sand to wetland; and wetland to lake (Figures 11 and 12). Felinks (2000) described plant succession in a post-mining landscape as having stages of stochastic regression instead of web-like stages of succession. Reverse succession in this context is defined as change from a particular LCT after a period of vegetation growth back to the initial or a characteristically comparable LCT via natural or artificial processes. Another notable difference in LC distribution between Schlabendorf Nord and Schlabendorf Süd was that, while in the North there was no recorded transition between open sand and pioneer grassland, in the Süd this transition was clearly visible. This suggests that vegetation growth in the Nord was not from the primary level as was the case with the Süd.

Also, while in Schlabendorf Nord, agriculture land played an essential role in forest growth and emerged as the most stable LCT because it was the most centrally located LCT in the ordination plane; in Schlabendorf Süd agricultural land was barely involved in forest growth, though it was the most stable LCT (Figures 11 and 13).

Unlike Schlabendorf Nord, the LC distributions in Schlabendorf Süd corresponded with the gradient of time.

### 4.2 Effect of land cover change on formation of lake in Schlabendorf post-mining landscape

**Figure 14:**
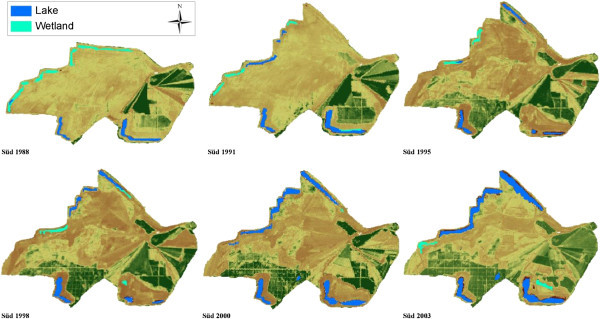
**Primary productions for Schlabendorf Nord and Schlabendorf Süd in 1988, 1991, 1995, 1998, 2000 and 2003.**

**Figure 15:**
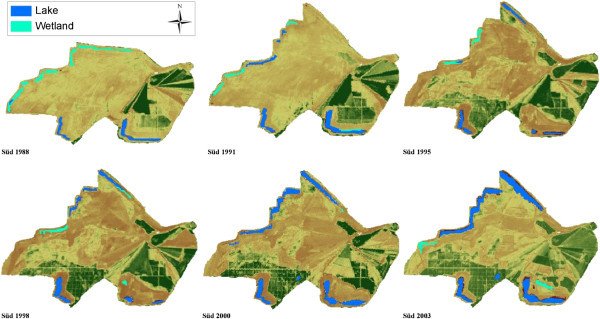
**Area of land occupied by lake in Schlabendorf Nord during 1988, 1991, 1995, 1998, 2000 and 2003.**

In Figures 14 and 15, areas of the post-mining landscape occupied by lake and wetlands are shown. Greater areas of water body in both post-mining landscapes were maintained (Figures 6 and 9). In Schlabendorf Süd there was constant increase in lake area during the study except in 1995. Since 1988, lake area has increased by four times its size (53.60 ha), with the most significant gain occurring between 2000 and 2003 (Figures 4, 5 and 14 and Table 2). Increase in lake size contributed to the reduced wetland area. However, only the first segment had a reduction in wetland area. A similar situation occurred in dry grassland and sparse pioneer grassland which also had a direct effect on reduced area of wetland. The ongoing transformation in lake and wetlands are mainly due to differences in age since dumping of spoils in the post-mining area, increased LU intensity and difference in water table levels in both landscapes. In Schlabendorf Nord, out of the 555.30 ha of land expected to become lake by 2010, about 300 ha had already formed lake as of 2003. However, only about 232 ha of the 659 ha of land expected to form lake by 2010 in Schlabendorf Süd had become lake in 2003 (Der Braunkohlenausschuss 1994). This can be explained by the fact that the old post-mining landscape (Schlabendorf Nord) has attained greater water table balance while the young post-mining landscape still needs some time to attain this balance. According to Grünewald (2001), the ‘greatest number of lakes in the Lusatia region will be filled in about 25 years’ time’. This could lead to fragmentation due to relocation of reclamation related roads and other construction activities. Following Grünewald’s finding and our observations, parts of dry vegetation, sparse pioneer grassland, dry grassland, open sand and wetland that are already changing to “lake” or share a direct boundary with “lake” would likely be affected by the increasing size of “lake”. The vegetation in these LUTs in both landscapes has a shallow fibrous root system or open sand which cannot serve as lake bank protection.

In order to operate an open cast mine, groundwater level should be pumped to beneath the deepest working level (Grünewald 2001). Since the mine closure in 1991 in Schlabendorf, pumping rate dropped, so that from 31.80 m^3^/s it reduced to 17 m^3^/s in 2000, leading to a rising groundwater table (Grünewald 2001), hence the significant increase in lake size in both landscapes.

The decreased lake size in 1995 could be due to changes in precipitation and high surface temperature. In August 1995 (satellite imagery acquisition date), the monthly average precipitation was below the mean value from 1961 to 1990. Furthermore, July 1995 was the hottest summer in the 15 years studied. Thus the reduced lake and wetlands area could be attributed to high surface temperature and dry summer conditions (Deutscher Wetterdienst).

Change in wetlands revealed a remarkable difference in LCC in both landscapes. Figure 15 shows wetland was mostly absent in Schlabendorf Nord. On the contrary, Schlabendorf Süd always had large areas of wetlands, with some measuring six times the largest wetland in Schlabendorf Nord (Figures 14 and 15). Thus the young post-mining landscape has an unstable groundwater table compared to the rather stable older landscape. This is the reason for the constant occurrence of wetlands in Schlabendorf Süd which in most cases turn into lake (Figure 14).

### 4.3 Biomass accumulation (Primary Production) in Schlabendorf

The afforested areas in the young landscape experienced a steady increase in primary production (about 25% pine and deciduous trees plantation) as intended in the reclamation goal (Bungart and Hüttl 2001). Furthermore, most of the afforestation of pine trees was maintained or increased continuously. This is reflected in the 44% biomass accumulation (Figure 12). Significant increase in mixed grassland also contributed greatly to succession successes and overall increase in biomass accumulation.

The fluctuating net biomass accumulated resulted from changing areas of dry grassland, dry vegetation, lake and open sand. NDVI values range from -1 to +1 but vegetation values typically fall within 0 to +1 with higher (greener) index values representing more active growth and primary production.

### 4.4 Effect of soil condition on land cover change in the post-mining landscape

**Figure 16:**
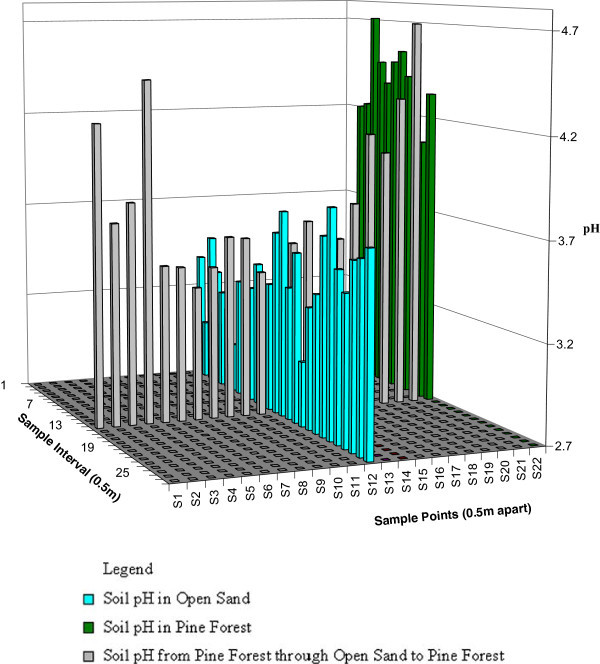
**Soil pH taken from failed pine afforestation area and successful pine afforestation.**

An important question that emerged was, why did some cultivated areas (e.g., afforestation of pine trees) turn into open sand? Incidentally, those failed afforested areas were along the path of a mine strip which probably had lower soil pH to support healthy plants. Soil pH in the area of failed afforestation of pine trees (along the path of the mine strip) were generally low (Figure 16), while those taken from the pine forest across the open sand areas were high on both sides of the pine forest but decreased in the open sand area. Samples taken strictly from the pine forest also had comparably high pH values.

Whenever soil becomes acidic some nutrients may become less available for plant uptake, although plants have different nutrient needs. Soil pH (CaCl2) of 5.20 to 8 provides suitable conditions for effective plant growth (Fernández and Eichert 2009); however, most soil pH recorded was above this range. High soil acidity such as that measure in Schlabendorf Süd could reduce or weaken populations and the activity of organisms that convert N, S, and P to plant-available forms. It became evident that low pH and impoverished soil condition were some of the causes of failed vegetation growth after afforestation in parts of the landscapes. Thus low soil pH and nutrient deficient soils contributed to reverse succession in Schlabendorf (Fernández and Eichert 2009).

### 4.5 Reclamation of mine-damaged areas in Schlabendorf

Quantification of the effects that mining activities have on ecosystems is a major issue in sustainable development and resources management (Latifovica et al. 2005). In Schlabendorf, the impacts of mining have spread beyond the site of extraction, affecting the neighbourhood of mined areas (Haigh 2000). These include constructed mining camps, roads, foot paths and contamination of nearby rivers (Spree River) by iron and soil or groundwater by acid. This situation requires some sort of restoration or intervention.

Parts of the post-mining landscape with greatly impoverished soils could be restored through some sort of human intervention (Bradshaw 1999; Hendrychová 2008; Groninger et al. 2007). However, reclamation of mine-damaged land is generally complex, often expensive and comes with specific problems based on the type of mining. In Schlabendorf, an estimated amount of 300 million Euros has gone into the reclamation program (Der Braunkohlenausschuss 1994). Bradshaw (1999) found out that reclamation schemes must focus on providing some sort of human intervention since succession may take a longer period. In Schlabendorf, such human interventions that could accelerate succession is the enrichment of the soil with nutrients before recultivation, especially in nutrient deficient areas that lost their vegetation cover. This is important since the topsoil was not replaced to re-establish soil fertility (Schultz and Wiegleb 2000). In such areas, enriching the topsoil through the application of selected diaspore enriched-heymulch could significantly facilitate primary succession. The addition of ashes (potassium) to the acidic soil is very vital to plant succession. It is worth noting that such an intervention measure is desirable in cases where the aim of reclamation is to get closed vegetation cover (e.g., towards protection from wind erosion), otherwise treatment should be restricted to areas which require them.

### 4.6 Causes of land cover changes in Schlabendorf

**Figure 17:**
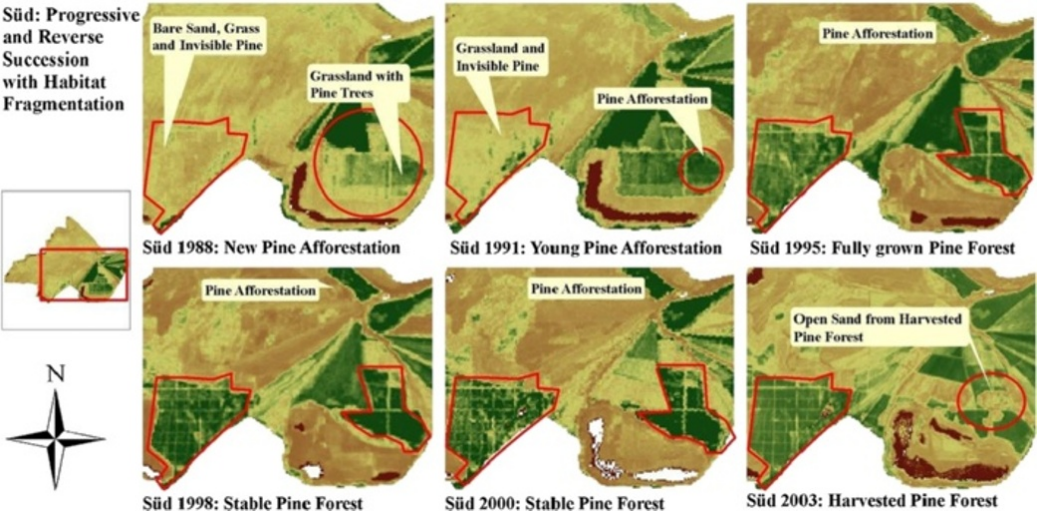
**Progressive and reverse succession in Schlabendorf.**

In order to understand the driving forces of future trends in LUC and the prospects for sustainability in post-mining landscape restoration, we need to identify the major driving forces. Causes of LCC can be direct and indirect, though a complex interaction of the two influences LCC at varying times and in different spaces (Geist et al. 2006). Nutrient deficient soil due to a turning of the upper topsoil, prolonged seasonal fluctuations in the microclimate, low soil pH (Haigh 2000), harvesting of afforested areas (Figure 17) and other restoration related construction activities was the leading cause of the LCCs and reversed succession in both landscapes (Bröring and Wiegleb 2005).

The process of LCC takes place at an interface between environmental and human systems interrelating with each other in a feedback mechanism (Geist et al. 2006; Demirel et al. 2011). And human policies such as “*stop pumping water from lakes”*, leveling of would-be-lake bottoms and soil compaction by explosives led to some of the observed LCC.

## 5 Conclusions

Visible patterns of increase and decrease in the LCT occurred in both landscapes. It is like a field laboratory where different habitat types interact to create new patterns of land cover types without any human societal influence such as population expansion, wealth generation etc.

Land cover transformations in the post-mining landscape showed similarity, yet it varied greatly in both landscapes. The variation reflects the impact of different ages of reclamation since dumping of spoils, land use intensity and difference in water table levels in both landscapes. Mixed grassland and tree in both landscapes was at the central point of the reclamation. Lower vegetation cover such as pioneer grassland, dry vegetation and dry grassland often change to mixed grassland and tree, which largely changed to the forest LCTs. In Schlabendorf Nord, afforestation often replaced afforestation of pine trees and deciduous tree afforestation. However, afforestation in Schlabendorf Süd was only replaced by afforestation of pine trees and mixed grassland and trees.

Though agricultural land emerged as the most stable LCT and played an essential role in forest growth, particularly in Schlabendorf Nord, agricultural land barely contributed to the afforestation of the post-mining landscape in Schlabendorf Süd.

LC transformations in the two post-mining landscapes subjected to different ages of reclamation since dumping are mostly the results of progressive and reversed plant succession. Nevertheless, the process of natural succession itself should be aided by human intervention if reclamation is to be accelerated.

Given two post-mining landscapes subjected to different ages of reclamation after dumping, clear differences in primary production and LCC pattern would occur. In Schlabendorf, the two post-mining landscapes are getting more diverse over time. The extent of these differences is partly due to the LU intensity and other geophysical factors. This is in line with the natural succession of an ecological process in an ecosystem.

During early stages of restoration, LCTs often have unstable conditions and experience more acute transformation due to increased LU intensity. This is eventually replaced by a more stable landscape with fewer transformations between the different LCTs.

Traditional mining operations like surface lignite mining as occurred within the Lower Lusatia region may not fit into the commonly understood definitions of sustainability. However, mineral extraction and later reclamation operations can contribute significantly to sustainability through the benefits they provide to society as well as the incorporation of sustainability principles in all phases of mining till its closure. This helps create a sustainable post-mining landscape suitable for predefined future LU after completion of reclamation activities and mine operation.

Land cover change takes place at the interface between environmental and human systems (Latifovica et al. 2005; Geist et al. 2006). Factors such as low soil pH, harvesting of afforested areas and other restoration related construction activities (Bröring and Wiegleb 2005) acted together or independently to bring about the LCCs.
